# Structure-Based Rational Design to Enhance the Solubility and Thermostability of a Bacterial Laccase Lac15

**DOI:** 10.1371/journal.pone.0102423

**Published:** 2014-07-18

**Authors:** Zemin Fang, Peng Zhou, Fei Chang, Qiang Yin, Wei Fang, Jing Yuan, Xuecheng Zhang, Yazhong Xiao

**Affiliations:** 1 School of Life Sciences, Anhui University, Hefei, Anhui, China; 2 Anhui Provincial Engineering Technology Research Center of Microorganisms and Biocatalysis, Hefei, Anhui, China; Russian Academy of Sciences, Institute for Biological Instrumentation, Russian Federation

## Abstract

Bacterial laccases are ideal alternatives of fungal laccases for specific industrial applications due to specific characteristics such as alkalescence dependence and high chloride tolerance. However, some bacterial laccases presented as inclusion bodies when expressing in *Escherichia coli* and showed thermal instability. In this study, rational design was employed to enhance the solubility and the thermostablity of the bacterial laccase Lac15-His_6_ based on the crystal structure obtained previously. After deletion of His-tag and residues^323–332^, the obtained Lac15D was completely expressed in soluble form even at a higher temperature of 28°C, compared to only 50% of Lac15-His_6_ expressed solubly at 16°C. It showed a two-time higher activity at temperatures lower than 35°C and a half-life increasing from 72 min to 150 min at 45°C. When used in chromogenic reactions, Lac15D showed constant activity toward dye precursors and their combinations under alkaline conditions, demonstrating its application potential in hair coloring biotechnology.

## Introduction

Laccases (benzenediol: oxygen oxidoreductase; EC 1.10.3.2), a family of multi-copper polyphenol oxidases, catalyze the oxidation of a certain range of inorganic and aromatic substances (particularly phenols), with the concomitant reduction of O_2_ to water [1]. In the presence of appropriate mediators, such as 2,2-azino-bis(3-ethylbenzothazoline-6-sulfonate) and 1-hydroxybenzotriazole, the substrate range of laccase can be further extended [2]. Because of their wide substrate range, requirement of no cofactors, and use of readily available oxygen as an electron acceptor, laccases were proved to have great commercial applications in cosmetics, pollutant detoxifications, pulp and paper industry, biotransformation and biotechnology [2], [3].

In nature, laccases are widely spread in the fungal and plant kingdoms, particularly in basidiomycetes [2], [3]. Unfortunately, nearly all fungal laccases lose their activities under alkaline conditions [4], [5], restricting their applications in modern biotechnical industries. Recently, laccases have been found to be widespread in bacteria [4], [6]. Of the reported bacterial laccases, several have been found to possess distinctive properties, including excellent activity and stability under alkaline conditions [4], as well as high halide tolerance [7], [8]. Compared with fungal laccases, the characteristics of bacterial laccases make them ideal alternatives for specific industrial applications such as hair coloring, as the coloring reactions are usually carried out at alkaline pH because the hair swells and the dye penetration is enhanced [4], [9].

The application of bacterial laccases needs large amounts of proteins with lower cost. However, a number of bottlenecks on the production of bacterial laccases exist. As *Escherichia coli* and pET vectors are the most frequently used prokaryotic expression combinations, a common limitation of recombinant bacterial laccase production is the formation of insoluble protein aggregates known as inclusion bodies [7], [10], [11]. Another obstacle is the thermostability of bacterial laccases. Although bacterial laccases such as CotA are thermally stable [12], other bacterial laccases reported today are temperature sensitive. For example, the laccase from *Stenotrophomonas maltophilia* only maintained 48% of its activity at 40°C and 25% at 50°C after 30 min of incubation; it was totally inactivated at 70°C [13]. All of these factors inhibit the application of bacterial laccases in industries.

Lac15 is a bacterial laccase screened from a marine metagenomic library and was successfully expressed in *E. coli* [7]. This bacterial laccase (called Lac15-His_6_ in this study) showed an excellent tolerance to halogen ion and had the ability to decolorize several industrial dyes of reactive azo class under alkaline conditions. However, the soluble expression temperature of Lac15-His_6_ must be kept at 16°C, and only about 50% of the expressed protein was soluble under this condition. Moreover, the half-life of Lac15-His_6_ was only 72 min at 45°C [7]. These would crucially limit the application of Lac15 in industries. To improve the application potential of Lac15, in this study, structure-based rational design was employed to enhance the solubility and the thermostablity of the protein. A Lac15-derived laccase, which was expressed in completely soluble form and was much more thermostable, was successfully obtained by removing the His-tag and the long flexible region. The application potential of the obtained laccase on chromogenic reactions, which are widely employed in hair colorization, was also evaluated. Our results show that the obtained mutational laccase a candidate in hair coloring biotechnology.

## Materials and Methods

### Strains, culture mediums, and chemicals

*E. coli* BL21 (DE3) was purchased from TransGen (Beijing, China). The strain was cultured in Luria–Bertani (LB) liquid medium. Syringaldazine was purchased from Sigma-Aldrich (St. Louis, MO, USA). Isopropyl-β-D-thiogalactoside (IPTG) and *Taq* DNA polymerase were purchased from TaKaRa (Dalian, China). All other chemicals and reagents were of analytical grade.

### Plasmids construction

Genes coding for two Lac15 derivatives (with or without histidine tag (His-Tag) at the C-terminal and called Lac15-His_6_ and Lac15, respectively; Fig. S1) were obtained by using the sequences listed in Table S1 as the primers and the previously reported *lac15* complete sequence as the template (GenBank accession No. HM623889). A two-step PCR method was used to construct a deletion mutant of *lac15* (*lac15*(-), the expression product is called Lac15D, Fig. S1). In a typical procedure, mutagenic oligonucleotide primer pairs of Lac15-FP and Lac15-D-RP, as well as Lac15-D-FP and Lac15-T-RP (Table S1), were used to amplify two DNA fragments, which were designated as S1 and S2, respectively. The two amplified DNA fragments were recovered. Using Lac15-FP and Lac15-RP as primers, and S1 and S2 as templates, PCR was then performed to amplify *lac15*(-). Compared with Lac15, residues ^323–332^ were deleted from Lac15D. Three expression plasmids were constructed as described using the expression plasmid pET22b(+) [7]. The recombinant plasmids were validated by sequencing. Each recombinant plasmid was individually transformed into *E. coli* BL21(DE3) to express Lac15 derivatives.

### Heterologous expression and purification of laccase proteins

When inducing the expression of proteins, *E. coli* BL21 (DE3) cells carrying recombinant plasmids were grown at 37°C in 200 mL LB medium containing 100 µg/mL ampicillin. IPTG at a final concentration of 0.2 mM was added into the culture of *OD*_600_  = 0.6. Immediately after adding of IPTG, the cultures were cultured at 16°C, 28°C, and 37°C for 10 h, 5 h, and 3 h, respectively. The cells were then collected by centrifugation. The pellets were resuspended in cold 20 mM Tris–HCl buffer (pH 8.0), disrupted by sonication, and then centrifuged at 30,000×g for 30 min.

The supernatants with soluble target proteins were applied to Hitrap Capto Q affinity chromatography (GE Healthcare, Piscataway, N.J., USA) to purify laccase proteins according to the manufacturer's instructions. Briefly, the column was eluted with a linear gradient of NaCl (0.1–1 M in 20 mM Tris-HCl buffer, pH 8.0, at a flow rate of 1.0 mL•min^???1^). The purified protein was dialyzed into a 50 mM Na_2_HPO_4_–KH_2_PO_4_ buffer (pH 7.5) and stored at 4°C.

### Sodium dodecyl sulfate–polyacrylamide gel electrophoresis (SDS-PAGE) and native-PAGE

The supernatants and precipitations of each induction group, and the purified laccase proteins were applied to SDS-PAGE to monitor the soluble expression of each protein and the purity of the purified proteins following the common procedure. Proteins were stained with Coomassie brilliant blue R-250. The purified laccase proteins were also applied to native-PAGE to determine the purity of the purified proteins. Activity staining of laccases was performed in 50 mM Na_2_HPO_4_–KH_2_PO_4_ buffer (pH 7.5) after PAGE using syringaldazine as the substrate. Protein concentration was determined using the BCA method (Bio-Rad, USA).

### Enzyme assay

The assay mixture consisted of 10 µL of appropriately diluted laccase protein and 990 µL of 50 mM Na_2_HPO_4_–KH_2_PO_4_ buffer (pH 7.5) containing 100 µM syringaldazine (*ε*_525_ = 65,000 M^−1^ cm^−1^) and 100 µM CuSO_4_. The reaction was initiated by adding enzyme to the solution. After incubation at 45°C for 5 min, the mixture was transferred into an ice-water bath for 30 s to stop the reaction, and the absorbance was measured at 525 nm [7]. Reactions with heat-treated laccase were used as the controls. One activity unit (U) was defined as the amount of laccase required to oxidize 1 µmol of substrate per minute.

### Laccase characterization

The pH effect on laccase activity was examined from pH 4.5 to 9.0 at 45°C in 50 mM sodium citrate buffer (4.5–5.5), Na_2_HPO_4_–KH_2_PO_4_ buffer (5.5–8.0), and Tris–HCl buffer (8.0–9.0). The temperature effect on the activity was measured by incubating laccase proteins within the temperature range of 20°C to 55°C at the optimum pH for the substrate. The enzyme stabilities against temperature were determined by incubating laccase at 45°C in a 50 mM Na_2_HPO_4_-KH_2_PO_4_ buffer (pH 7.5). The residual activities were then determined as mentioned above.

### Chromogenic reactions

When conducting the chromogenic reactions, *p*-phenylenediamine (PPD), *o*-phenylenediamine (OPD), *o*-aminophenol (OAP), 2,6-dimethylphenylalanine (DMP), and *p*-aminophenol (PAP) were dissolved in 10% DMSO solution (v/v). When inducing the reactions, the 100 mM Na_2_HPO_4_-KH_2_PO_4_ buffer (pH 6.5, 7.5, and 8.5); 20 U/L Lac15D, and substrates were mixed in the 96 well plates. The total volume of the reaction mixture was 100 µL, whereas the final concentration of the substrate, buffer, CuSO_4_, and DMSO was 5 mM, 0.1 M, 100 µM, and 1% (v/v), respectively. Reaction mixtures were incubated at 40°C for 3 min, and then the oxidation was monitored spectrophotometrically according to Saito [9].

## Results and Discussion

### Structure-based rational design of Lac15

**Figure 1:**
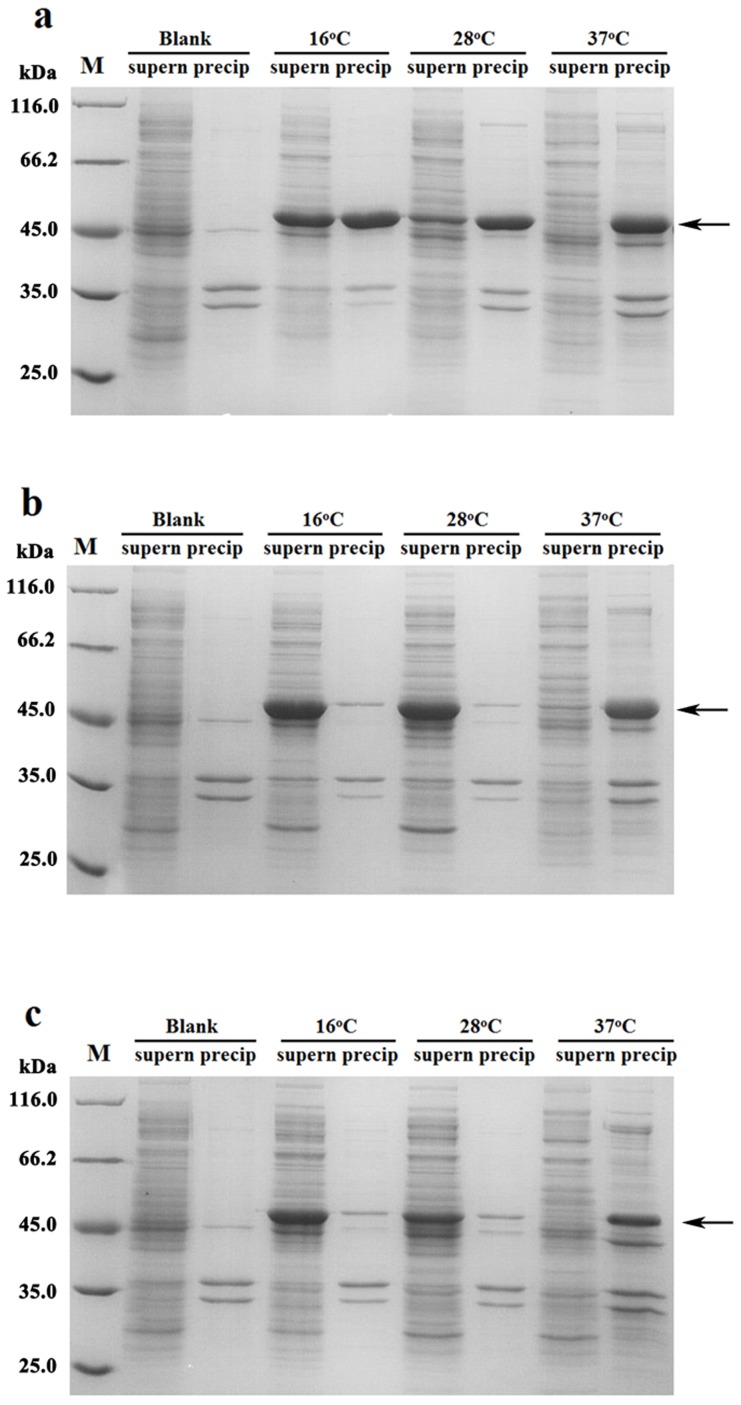
SDS-PAGE of (a) Lac15-His_6_, (b) Lac15, and (c) Lac15D under different induction temperatures.

Since the first report on bacterial laccase from *Azospirillum lipoferum*, a few bacterial laccases have been cloned and recombinantly expressed in *E. coli* in recent years [4]. Several of them were expressed as inclusion bodies [7], [10], [11]. Stress conditions and high rates of protein synthesis are believed to be the main causes of the formation of inclusion bodies [14]. As a result, a few fermentation parameters have been modulated to enhance soluble protein expression. For example, cultivation at decreased temperatures to decrease the in vivo aggregation of recombinant polypeptides is prevalent and effective [10]. Our previous results also indicate that lower temperature can enhance the soluble expression of bacterial laccase [7]. However, only approximately 50% of expressed protein was soluble after a range of fermentation condition optimization processes (Fig. 1a), indicating that some other factors may affect laccase folding. In addition, the purified recombinant Lac15-His_6_ was rather unstable, with only 50% of the initial activity retained after 72 min of incubation at 45°C [7]. These disadvantages would hamper the application of Lac15 in industries.

**Figure 2:**
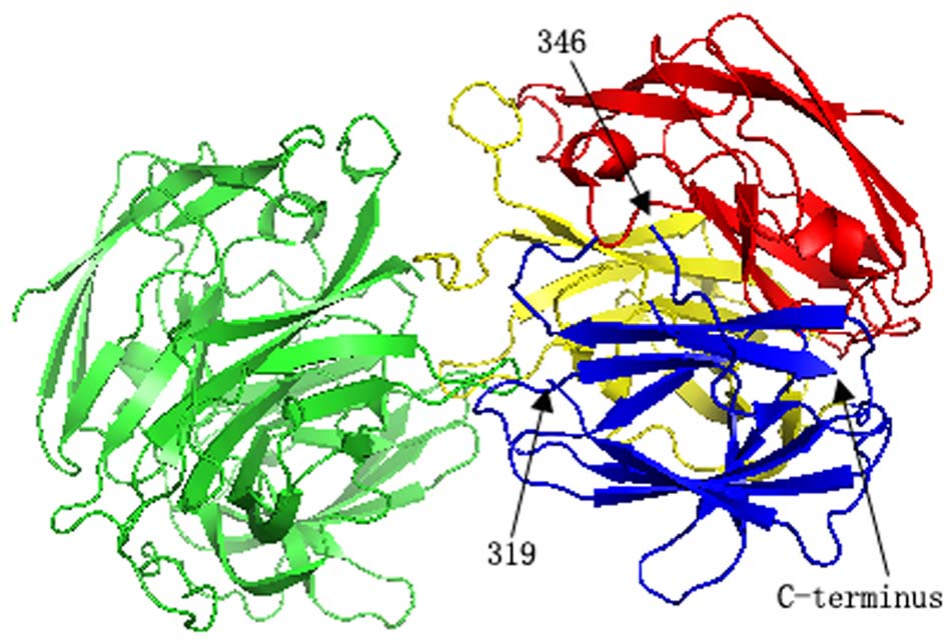
The carton illustration of the crystal structure of C-terminally His-tagged Lac15 (PDB No. 4F7K). The structure is composed of a homogenous dimer, in which one molecule is shown in green, and the three cupredoxin-like domain of the other are shown in red, yellow and blue respectively. The arrows point to the start and the end of the longest invisible region and the visible C-terminus of the protein structure (the last but one residue of Lac15 sequence connected with the His-tag).

Some structural factors may affect the solubility and thermostability of the proteins. Thus, to enhance the yield and the duration of the recombinant Lac15 through rational design, the structure of Lac15-His_6_ was resolved using X-ray crystallography method [15]. The crystal structure of Lac15-His_6_ (PDB No. 4F7K) adopts a typical fold of a bacterial laccase, which comprises three cupredoxin like domains formed by multiple-stranded Greek key β-barrel (Fig. 2). Notably, the extreme C-terminus of the Lac15, which is immediately followed by the His-tag, is an ordered β-strand. In addition, a few regions, including the C-terminal His-tag, are invisible in the electronic density map, indicating high flexibility within these areas. As His-tag sometimes would affect the solubility of the proteins by interfering with the correct fold of the proteins, the formation of the inclusion body of Lac15-His_6_ might result from the impact of the flexible His-tag on the structure of its preceding β-strand. On the other hand, the flexible regions in proteins, especially the longest one i.e. residues 320–345, would reduce the thermostability of Lac15, as they might enhance the entropy during protein unfolding by increasing the numbers of unfolded conformations that can be sampled by the protein [16]. Thus, remove the His-tag and delete the residues 320–345 would hopefully promote the solubility and thermostability of Lac15, as in the cases of other proteins [17], [18], [19]. To illustrate it and produce Lac15-derived laccase with improved yield and thermostability, the wild-type Lac15 containing none tag and its mutant with the residues 323–332 removed were recombinantly expressed, purified and characterized.

### Effects of the His-tag on the solubility of Lac15

After deleting the His-tag, Lac15 was completely expressed in soluble form when induced at 28°C (Fig. 1b). By contrast, about 15% of the soluble protein was obtained in His-tag-containing laccase when induced at 28°C (Fig. 1a). Two proteins were presented in the form of inclusion bodies when induced at 37°C. The results demonstrated that the His-tag, as we speculated, is critical for the formation of inclusion body of Lac15-His_6_ when expressed at temperatures lower than 28°C. The completely soluble expression of laccase protein at near room temperature will decrease the costs of enzyme production and saves energy on maintaining the lower temperature in fermentation systems.

His-tag is the most popular fusion tag used in recombinant protein biotechnology. It facilitates the purification and detection processes of recombinant proteins [20]. Since the first description of purifying proteins with histidine residues, thousands of industrial enzymes and pharmaceutical proteins have been expressed and purified [20], [21]. They have also been used to eliminate the variability in expression yields. In some cases, His-tags have been observed to prevent the formation of inclusion bodies [22]. In our study, however, based on the immediate linkage between the flexible His-tag and the ordered C-terminus in the crystal structure of Lac15-His_6_, His-tag was evaluated as the main reason for fold disturbation and inclusion body formation. Similar cases have been reported by Li [18] and Zhu [23], who found that removing the fusion His-tag can promote the solubility of heterologous proteins.

### Effect of flexible region on the thermostability of Lac15

**Figure 3:**
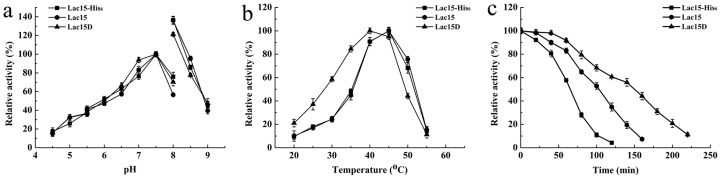
Effects of pH and temperature on the activity and stability of laccase proteins. (a) pH optimum. Laccase activity was determined in 50 mM sodium citrate buffer (4.5–5.5), Na_2_HPO_4_–KH_2_PO_4_ buffer (5.5–8.0), and Tris–HCl buffer (8.0–9.0). (b) Temperature optimum. Laccase activities were measured by incubating proteins at each optimal pH. (c) Temperature stability. Proteins were incubation at 45°C at pH 7.5. The data presented are the average values from triplicate technical repeats of measurements.

Highly flexible regions represent an important part of the protein structure and have been found to be important in protein function, stability, and folding [24]. In the past decades, the decrease in the flexibility of highly flexible regions has been proven to enhance the thermostability of enzymes such as D-xylose isomerase [25] and α-glucosidase [26]. As a result, the amino acid residues that constitute the flexible region of 320–345 were deleted step-by-step. When residues^323–332^ was deleted from Lac15, the resulting Lac15D exhibited significantly improved thermostability. The half-life of Lac15D at 45°C was 150 min, increased by nearly half compared to Lac15, which showed a half-life time of 102 min at 45°C (Table 1 and Fig. 3c). The enhanced thermostability caused by deletion was also supported by the modeled structure of Lac15D, which presented a short helix, rather than a disordered conformation (data not shown), within the region corresponding to the invisible region in Lac15-His_6_ (PDB No.4F7K).

**Table 1:** Substrate specificity of Lac15, Lac15-His_6_, and Lac15D.

Protein	*K*_m_ (µmol·L^−1^)	*k*_cat_ (s^−1^)	*k*_cat_/*K*_m_ (×10^5^)	Specific activity (U·mg^−1^)	Optimal temperature (°C)	T_1/2_(45 °C) (min)
Lac15-His_6_	4.53±0.21	1.35±0.09	2.98±0.18	1.15±0.08	45	72
Lac15	3.74±0.37	1.39±0.11	3.72±0.21	0.83±0.06	45	102
Lac15D	6.34±0.19	1.69±0.13	2.67±0.14	1.10±0.11	40	150

However, our further investigation indicated that enhanced thermostability of Lac15-D might not be due to decreased flexibility in the shortened loop. We have resolved the crystal structures of non-tagged Lac15 and Lac15-D, both of which display little difference when compared with that of Lac15-His_6_ (data not shown). Notably, the fragment invisible in the crystal structure of Lac15 is missed in Lac15-D as well, which means the shortened loop is still significantly flexible. In addition, using the maximum emission wavelength of intrinsic Trp fluorescence as probe, we measured the thermodynamic stability of non-tagged Lac15 and Lac15-D against denaturant GdnCl, and the result showed that the two proteins are indistinguishably stable (data not shown). Thus, there must be some other mechanisms through which the flexible region contributes to the thermostability of Lac15. Its elucidation needs more in-depth investigation.

Lac15 also showed improved thermostability compared to Lac15-His6, showing that His-tag also affects the thermostability of Lac15-His6. As indicated above, the attachment of the His-tag at the end of C-terminus would interfere with the protein conformation. This scenario may decrease the interaction forces such as the hydrogen bond and hydrophobic effect between the residues in Lac15-His6, cause its sensitivity to temperature compared with Lac15, and finally cause physical inactivation at a shorter time.

### Comparison of the biochemical characterizations of Lac15 derivatives

**Figure 4:**
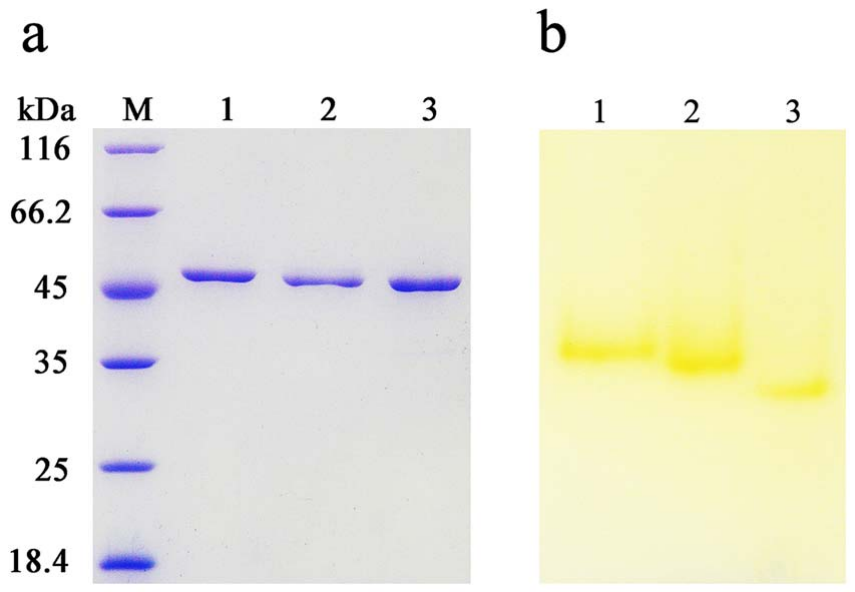
Purified Lac15-His_6_ (lane 1), Lac15 (lane 2), and Lac15D (lane 3) assayed by (a) SDS-PAGE and (b) native-PAGE. Activity staining of laccases was performed in 50_2_HPO_4_–KH_2_PO_4_ buffer (pH 7.5) after PAGE using syringaldazine as the substrate.

The purified proteins exhibited a single band both on SDS-PAGE (Fig. 4a) and native-PAGE (Fig. 4b), suggesting a homogenous form. As a result, these proteins were used for further research. Lac15 and Lac15-His_6_ share similar catalytic properties such as the same optimal pH values (Fig. 3a) and optimal temperatures (Fig. 3b). When residues^323–332^ were deleted from the protein, the obtained Lac15D showed a lower optimal pH compared with these two proteins, with an optimal temperature of 40°C (Fig. 3b). Lac15D also showed twice higher catalytic activity at temperatures lower than 35°C. For example, it retained approximately 80% and 60% of the original activities at 35°C and 30°C, respectively. However, Lac15 and Lac15-His_6_ only retained approximately 40% and 30% of the activities under the relevant temperatures, respectively (Fig. 3c). Usually, the decrease in flexibility would enhance the optimal temperature and the thermal stability of the proteins [27]. The deletion of residues^323–332^ of Lac15 resulted in lower optimal temperature and better cold adaption might indicate that this region plays a more sophisticated role in the protein structure and function.

His-tag fusion has little effect on the properties of the proteins except for the solubility. Consistent with this, Lac15 and Lac15-His_6_ showed similar substrate affinity and catalytic rate towards the substrate syringaldazine, with a *K*_m_ value of 3.74×10^−6^ M and 4.53×10^−6^ M, and a *k*_cat_ value of 1.39 s^−1^ and 1.35 s^−1^, for the former and the latter respectively (Table 1).

The flexible region plays important roles in the structure and function of the proteins. Actually, the *K*_m_ value of Lac15D was 6.34×10^−6^ M, higher than that of Lac15, indicating that the deletion of the residues 323–332 decreased the substrate affinity of Lac15. Meanwhile, the *k*_cat_ of Lac15D was 1.69 s^−1^, higher than that of Lac15, indicating that the deletion promoted the catalytic rate (Table 1). As the loop where residues^323–332^ located is near the the substrate entrance to Cu I (Fig. 2), its reduction might impact the binding of substrates, which leads to higher *K*_m_. Again, the inconsistence between the effects of the deletion on the *K*_m_ and *k*_cat_ might imply the sophisticated roles of the region in the protein structure and function. To elucidate it, further in-depth structural investigation on the region and the protein was needed.

### Chromogenic reactions

One possible application of laccase is in hair coloring [9], [28]. Commercial oxidation hair color usually consists of dye precursors and an oxidizing agent. The dye precursors usually contain *p*-diamines, PAP, and some other phenols to produce various colors. After adding an oxidizing agent such as H_2_O_2_, they form chromatic indo dyes [9]. However, side reactions with the hair proteins occur simultaneously because of the severe reaction condition and can cause hair damage [29]. If H_2_O_2_ is replaced by another oxidizing agent such as laccase, the hair damage should be less. Unfortunately, the coloring reactions are usually carried out at alkaline pH because the hair swells and the dye penetration is enhanced. As a result, laccase should have constant activity under an alkaline condition.

**Figure 5:**
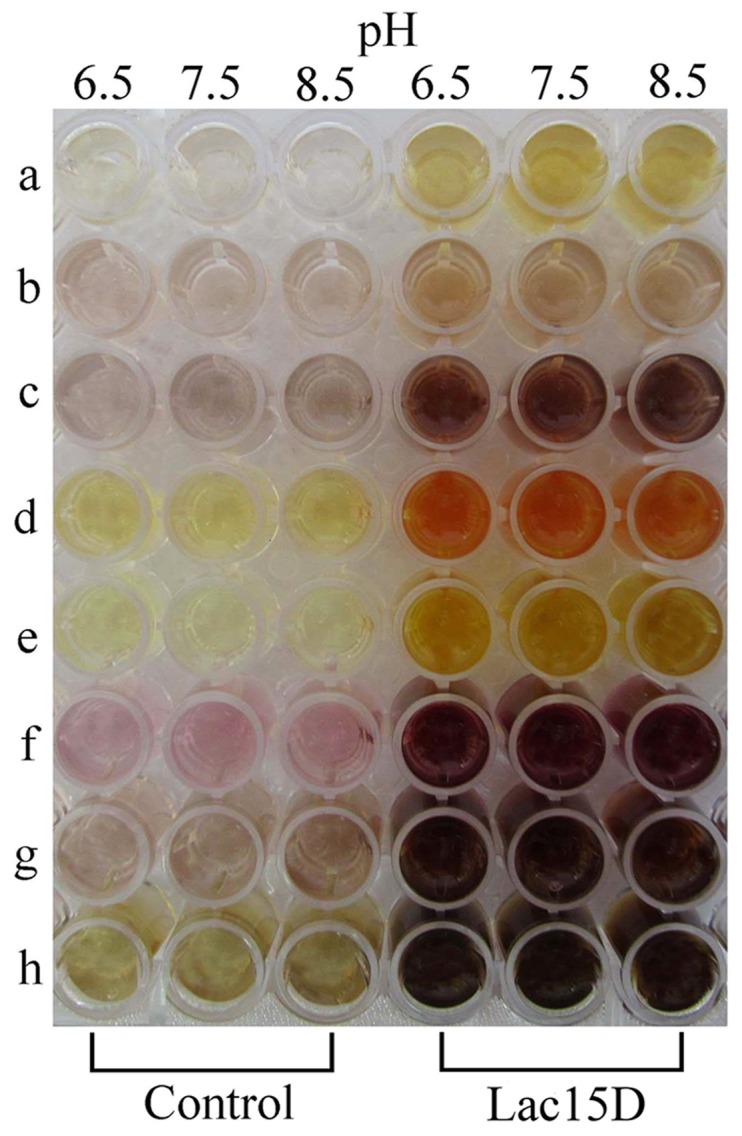
The result of the chromogenic reaction of Lac15D for the dye combination. (a) DMP. (b) OAP. (c) PPD. (d) PAP. (e) OPD. (f) PPD and DMP. (g) PPD and OAP. (h) PPD and PAP.

Based on the biochemical characterization results, the optimal catalytic pH in alkaline condition retained approximately 60% of the original activity at 30°C and showed thermostability at temperatures lower than 45°C. The application potential of Lac15D on hair coloring was investigated, and dye precursors such as PPD, MPD, OAP, DMP, and PAP were used as the representatives. The chromogenic reaction of Lac15D for the dyes is shown in Fig. 5. Lac15D showed oxidation activity for 5 dye compounds at pH 6.5, 7.5, and 8.5, and exhibited higher activities toward DMP and PAP. These results are the same as laccase from *Thermobifida fusca*, which also displayed better activity toward DMP and PAP [30]. In contrast, the oxidation effect of laccase was less for OAP (Table 2).

**Table 2:** Oxidation activity of Lac15D for PPD, OPD, OAP, DMP, and PAP under different pHs.

Substrate	Wave length nm	Absorbance		
		pH 6.5	pH 7.5	pH 8.5
*p*-phenylenediamine	470	0.64±0.17	0.533±0.10	0.42±0.12
2,6-dimethoxyphenol	470	1.21±0.21	1.38±0.27	1.95±0.20
*o*-phenylenediamine	430	0.54±0.14	0.41±0.08	0.41±0.11
*o*-aminophenol	450	0.28±0.07	0.33±0.12	0.35±0.15
*p*-aminophenol	405	0.71±0.11	0.95±0.26	0.89±0.14

The application potential of laccase in the hair coloring process has been proposed in literature. However, some applications have not been realized. Saito investigated the oxidation activities of laccase Flac1 from *Flammulina velutipes*, bilirubin oxidase from *Myrothecium verrucaria*, and laccase RvL from *Rhus vernicifera* toward dye precursors. Their activities shifted depending on the pH [9]. For example, the activity of Flac1 was higher at pH 6.5 than at pH 9.0 for all tested compounds. In contrast, the Lac15D activity toward 5 dye precursors did not apparently change at the pH range of 6.5 to 8.5 (Fig. 5 and Table 2), which may overcome the shortage of shifted optimal pH according to the substrates in alkaline laccases and validate the potential application of Lac15D in hair coloring industries.

## Conclusion

Structure-based rational design was employed to enhance the solubility and the thermostablity of the bacterial laccase Lac15-His_6_. By removing the His-tag and the residues^323–332^, the obtained Lac15D showed a completely solubility, a higher thermostability, and a better cold adaption compared to Lac15-His_6_. Lac15D showed a constant catalytic activity towards dye precursors and their combinations, which are widely used in hair color, under alkaline conditions, indicating Lac15D a candidate in hair coloring biotechnology.

## Supporting Information

Figure S1 **Construction of different genes in the pET22b(+) plasmid and the proteins expressed by these constructs.** Lac15-His_6_, single His-tag fusion at the C-terminus. Lac15, protein without any His-Tag. Lac15D, residues of 323–332 were deleted from Lac15.
(TIF)

Table S1 Primers used in plasmids construction steps.
(DOCX)
