# Supplementary figures and images for: Structure-Based Rational Design to Enhance the Solubility and Thermostability of a Bacterial Laccase Lac15
Source: PLoS One. 2014 Jul 18;9(7):e102423. doi: 10.1371/journal.pone.0102423 (PMC4103834; doi:10.1371/journal.pone.0102423)

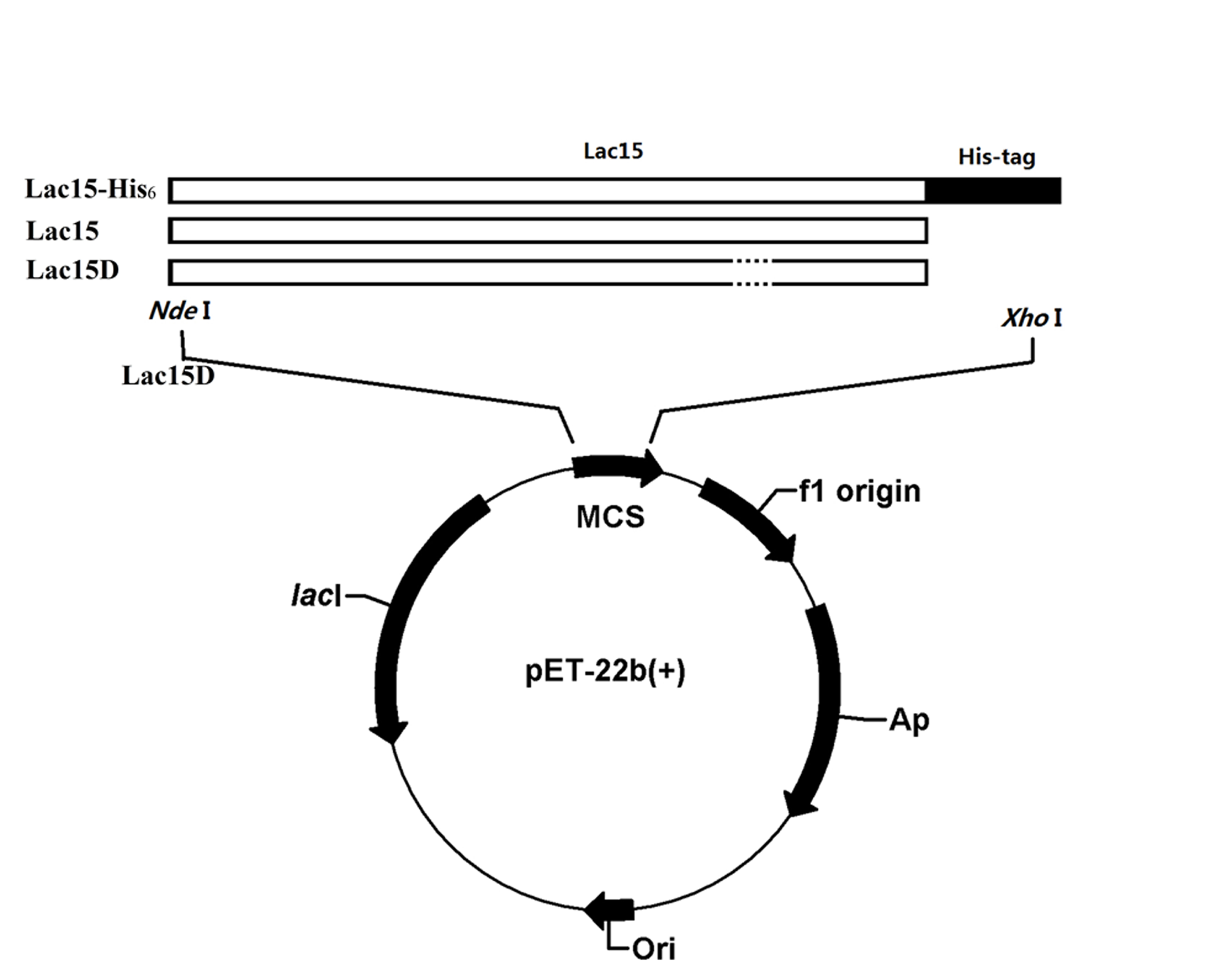

Supplement: Figure S1 — Construction of different genes in the pET22b(+) plasmid and the proteins expressed by these constructs. Lac15-His6, single His-tag fusion at the C-terminus. Lac15, protein without any His-Tag. Lac15D, residues of 323–332 were deleted from Lac15. (TIF) [file pone.0102423.s001.tif]
