# Supplementary material for: Structure-Based Rational Design to Enhance the Solubility and Thermostability of a Bacterial Laccase Lac15
Source: PLoS One. 2014 Jul 18;9(7):e102423. doi: 10.1371/journal.pone.0102423 (PMC4103834; doi:10.1371/journal.pone.0102423)
Supplement: Table S1 — Primers used in plasmids construction steps. (DOCX) [file pone.0102423.s002.docx]

**Table S1**

| **Primer** | **Sequence (5’→3’)** | **Restriction enzyme** | **Description** |
| --- | --- | --- | --- |
| Lac15-FP | GGAATTC*CATATG*AGCGCACCGGTTGAACTG | *Nde*I underlined | Remove the N-terminal signal peptide |
| Lac15-T-RP | CCAG*CTCGAG*TTATGCAACTTCAACCCAGGTTTTC | *Xho*I underlined | Remove the C-terminal His-tag |
| Lac15-RP | CCAG*CTCGAG*TGCAACTTCAACCCAGGTTTTC | *Xho*I underlined | Carry the C-terminal His-tag |
| Lac15-D-FP | TGACCCTGACCATGGAAGGTATGATGGGTGGCGATATTTG | ― | Delete 10 residues (323-GAMSRRMMQG-332) |
| Lac15-D-RP | CAAATATCGCCACCCATCATACCTTCCATGGTCAGGGTCA | ― |  |
